# High-altitude mountaineering induces adaptive gut microbiome shifts associated with dietary intake and performance markers

**DOI:** 10.1038/s41598-025-22848-9

**Published:** 2025-10-27

**Authors:** Ewa Karpęcka-Gałka, Kinga Zielińska, Barbara Frączek, Paweł P. Łabaj, Tomasz Kościółek, Kinga Humińska-Lisowska

**Affiliations:** 1 Doctoral School of Physical Culture Sciences, University of Physical Culture in Krakow, Krakow, Poland; 2 Malopolska Centre of Biotechnology, Jagiellonian University, Krakow, Poland; 3 Department of Sports Medicine and Human Nutrition, Institute of Biomedical Sciences, University of Physical Culture in Krakow, Krakow, Poland; 4 Sano Centre for Computational Medicine, Krakow, Poland; 5 Faculty of Physical Culture, Gdansk University of Physical Education and Sport, Gdansk, Poland

**Keywords:** Gut microbiome, High-altitude mountaineering, Dietary intake, Performance markers, Metagenomics, Physiological adaptation, Bioinformatics, Microbiology techniques, Diagnostic markers, Physiology, Health care

## Abstract

This study examined how high-altitude exposure and expedition-specific dietary changes influence gut microbiome composition, functional pathways, and their relationships with performance and health markers in alpinists. Seventeen male mountaineers (age 30.29 ± 5.8 years) participating in multi-week expeditions (> 3,000 MASL) were assessed before and after their climbs. Assessments included dietary intake analysis, blood and urine biomarkers, aerobic and anaerobic performance tests, and metagenomic sequencing of the gut microbiome. Bioinformatic and statistical analyses evaluated changes in microbiome composition and function and their correlations with physiological and dietary parameters. High-altitude exposure was associated with significant shifts in gut microbial composition and functional capacity. While the total number of bacterial species and functions remained stable, the glucose degradation pathway increased post-expedition. Participants with greater microbiome shifts showed improved performance and had richer baseline microbiomes. Pre-expedition, certain microbial functions were associated with vitamin B₆ and C intake, while post-expedition correlations involved specific macronutrients and micronutrients. Additionally, some microbiome changes correlated with blood markers, indicating links to nutrient metabolism and electrolyte balance. The gut microbiome of alpinists adapts to extreme environmental stress and dietary changes, influencing metabolic, immune, and performance-related processes. Optimizing dietary strategies to support a beneficial microbiome profile may enhance resilience and performance in challenging high-altitude environments.

**Supplementary Information:**

The online version contains supplementary material available at 10.1038/s41598-025-22848-9.

## Introduction

 During climbing at high altitudes (> 2,500 MASL), alpinists must deal with reduced atmospheric pressure, leading to the development of hypobaric hypoxia, intense solar radiation, variable weather conditions, strong winds and low temperatures with large fluctuations throughout the day, as well as the risk of avalanches and falling rocks^1,2^. During high-altitude expeditions, dietary changes occur due to poor access to fresh food, lack of appetite, food poisoning, harsh environmental conditions, and physiological changes^3,4^. Reactive oxygen species (ROS) production is accelerated with mountainous elevation, which may play a role in developing serious health crises. Exposure to increasing altitude leads to a reduction in ambient O_2_ availability in cells, contributing to hypoxic oxidative stress and disturbed redox homeostasis, potentially impairing exercise performance and recovery^5,6^. As a result of increased ROS production, alpinists may experience cognitive decline, development of neurodegenerative processes, pathological changes in brain structures, including vasogenic edema^7^, as well as damage to the intestinal barrier. This intestinal barrier disruption may lead to bacterial translocation and local or systemic inflammatory reactions, further impacting both general health and physical performance^8^. A damaged intestinal barrier may impair absorption of nutrients, which could explain some of the weight loss observed during prolonged exposures to high altitude^9,10^.

In addition to these physiological stresses, blood parameters and body composition can be altered by high-altitude conditions and changes in dietary intake. This may affect red blood cell counts, iron status, and markers of metabolic and immune function^11–15^. Similarly, adjustments in macronutrient and micronutrient intake before and during the expedition can affect an athlete’s energy availability, leading to shifts in body mass and composition^16^, and potentially affecting maximum oxygen uptake (VO_2max_), anaerobic power, and other key performance indicators. Such high-altitude exposures thus serve as a natural experimental setting to assess how nutrition, physiology, and microbial ecology interact to shape athletic performance, health status, and adaptation to extreme environments.

In recent years, the gut microbiome has emerged as a critical factor influencing human health, immune function, and athletic performance. Alterations in gut microbial composition and metabolic capacity have been linked to changes in nutrient absorption, energy metabolism, inflammation, and recovery, all of which are central to optimal athletic performance and well-being^17–19^. In addition, the gut microbiota can respond dynamically to dietary changes, and high-altitude expeditions often require consumption of processed, freeze-dried, or supplemental foods due to limited access to fresh produce^20^. The use of functional foods has important advantages for athletes, such as the fact that they can be easily ingested, transported and stored, and they also taste good. However, when consumed in excess, these products may disrupt gut microbiota balance due to the chronic effects of their food additives^21–24^. Understanding how these dietary and environmental stresses shape gut microbial communities is essential for informed nutritional strategies that support health, performance, and safe acclimatization to challenging conditions.

The present study aimed to analyze the structure and functional characteristics of the microbiome of climbers before and after a high-mountain expedition. By integrating detailed dietary records, blood and urine analyses, and measures of aerobic and anaerobic exercise capacity, we sought to determine how high-altitude exposure and associated dietary changes affect the gut microbiome and, in turn, how these microbial shifts correlate with health markers and performance outcomes. Such insights could provide evidence-based guidance for optimizing dietary composition, supplementation, and overall expedition planning. Ultimately, identifying specific microbial signatures and metabolic pathways that are modifiable through diet and supplementation may help practitioners develop tailored nutritional interventions to improve both health and athletic performance of climbers operating under extreme environmental conditions.

## Materials and methods

### Study participants

The study group consisted of 17 male mountaineers from Poland between the ages of 23 and 40, participating in mountain expeditions at least once a year, during which they stayed at altitudes above 3,000 m for at least 3 weeks. Individuals qualified for the study were members of high-mountain clubs affiliated with the Polish Mountaineering Association. The group’s anthropometric and BMI (body mass index) data are shown in Table 1.

**Table 1:** Anthropometric data and BMI of climbers participating in the research (*n* = 17).

	Men (*n* = 17)	
	value	SD
Age [years]	30.29	5.8
Body height [cm]	180.47	8.36
Body weight [kg]	74.96	5.03
BMI [kg/m^2^]	22.83	2.1

Abbreviations: BMI - body mass index.

The climbers participating in the study were characterized by extensive climbing experience (10 ± 5 years) in both sport climbing and mountaineering. The average climbing level of participants, as measured by the IRCRA scale, was 20.69 ± 2.8, which according to the proposed criteria, places them in the advanced (level 3) category^25^. The climbers spent an average of 8 ± 3 h per week training. Climbers qualified for the study stayed at an altitude of 3,000–8,167 m above sea level at the time of their expedition. In total, the high-altitude expeditions lasted an average of 34 ± 6 days, of which climbers spent an average of 22 ± 4 days actively climbing in the mountains and 11 ± 8 days resting in base camps or mountain towns. Because some of these sites were located above 3,000 m, participants spent a total of 26 ± 6 days at altitudes above 3,000 m.

The mountaineers recruited for the study traveled in small groups or individually to pursue their mountain goals. Specifically, the objective of the group of climbers (*n* = 6) exploring the White Cordillera massif in Peru was to climb the 800-meter Cruz del Sur route on the La Esfinge rock monolith (5,325 m). A new route was also established on Ocschapalca (5,888 m) and Nevado Churup (5,495 m), and ascents were made on Artesonraju (6,025 m) and Alpamayo (5,947 m). Climbers (*n* = 3) targeting peaks from the Shuijerab mountain group in North Karakorum, Pakistan, made a route on the west face of the virgin peak of Trident Peak (6,150 m), and climbed the virgin peak of Sakwa Sar (6,050 m). Climbers (*n* = 3) exploring the Gangotria Valley in India’s Garhwal Himalaya and mountaineers (*n* = 2) climbing in the Himalayas in Western Nepal and Northern Nepal attempted virgin peaks, but failed due to weather conditions (the highest point of the expeditions − 5,000 m). There were also two independently active climbers in the Himalayas - one of them made ascents of Annapurna (8,091 m) and Dhaulagiri (8,167 m) in Central Nepal, while the other climbed Ama Dablam (6,812 m). In the western Pamir-Alay in Kyrgyzstan’s Lailak Valley, an ascent was made by the Troschenko route on the north face of Ak-su (5,217 m).

Before taking part in the project, the climbers had a consultation with a sports medicine doctor. The climbers’ fitness for mountaineering and participation in the project was assessed based on previous examinations (ECG, blood and urine tests). The presence of chronic diseases and age over 45 were disqualifying factors for participation in the study. The Bioethics Committee of the Cracow Regional Medical Chamber approved the research project (68/KBL/OIL/2022; date: 11/04/2022), which was conducted in accordance with the Declaration of Helsinki. After learning about the risks and benefits of taking part in the project, all climbers gave written informed consent to participate in this study.

## Study design

### Nutritional analysis of diet

The supply of selected macronutrients before and during the mountaineering expedition was determined by analyzing the whole-day rations obtained using the 3-day food diary method. The expedition participants were asked to note down all the foods, meals and supplements eaten and fluids drunk during the 3 days before the expedition (2 days of sports activity, 1 day of rest) and 3 days during the expedition (2 days of sports activity, 1 day of rest). In the mountain conditions, these were days of acclimatization and ascending towards the summit, and the activities noted during this time included mainly hiking with elements of climbing. Climbers were given detailed instructions on how to keep food diaries before leaving for the expedition in order to minimize recording errors. The instructions included written guidelines (the table of household measures of popular foods with equivalent weights to make it easier to determine product measures and an example of a completed diary) along with a briefing session explaining how to record all foods, beverages, and supplements consumed. Participants were asked to specify product names, manufacturers, quantities (in grams, milliliters, or standard household measures), preparation methods (if applicable), and the time of consumption. Special attention was given to recording commercially packaged products such as energy gels, bars, and freeze-dried meals, for which climbers were instructed to note the exact product name, brand, and weight to allow analysis based on the product’s label data and nutritional table.

The data from the diaries were meticulously entered into the Aliant Dietetic Calculator program (Anmarsoft, Gdańsk, Poland; version: 85; database: 6.2) for quantitative analysis of the climbers’ diets before and during the expedition. The program used the “Tables of food composition and nutritional value”^26^ as a database, taking into account food recipes and losses due to product processing.

### Anthropometric measurements

Body height of participants was measured before the expedition using a Seca 217 anthropometer (Seca GmbH & Co. KG, Hamburg, Germany) with an accuracy of 1 mm. The climbers’ body weight was determined using InBody 120 body composition analyzer (Inbody Bldg., Seoul, Korea) in the morning after a standardized meal between 7:45 and 8:30 am.

### Health status analysis

For health status analysis, blood samples were taken from veins in the elbow pit to determine hematological and biochemical blood indices, i.e. peripheral blood count (hematocrit, hemoglobin concentration, erythrocyte volume (MCV), erythrocyte hemoglobin content (MCH), erythrocyte hemoglobin concentration (MCHC), erythrocyte anisocytosis index (RDW), erythrocyte count, platelets, platelet volume (MPV), white blood cell count - leukocytes, leukocyte differentiation: lymphocytes, monocytes, basophils, eosinophils, neutrophils), erythrocyte sedimentation rate (ESR), glucose, total cholesterol, high-density lipoprotein cholesterol (HDL), low-density lipoprotein cholesterol (LDL), triglycerides (TG), creatinine (Cr), urea (U), uric acid (UA), total protein, sodium, potassium, chloride, calcium, magnesium, phosphorus, iron, ferritin, vitamin D, vitamin B_12_, liver enzymes: alanine aminotransferase (ALT), aspartate aminotransferase (AST), acid phosphatase, gamma-glutamyltranspeptydase (GGTP), albumin and total bilirubin. The determinations were carried out in the morning and on an empty stomach, up to two weeks before the expedition and within two days (1.41 ± 0.69 days) after the expedition.

In addition to the blood determinations, a general urinalysis (specific gravity, pH, leukocytes, nitrites, protein, glucose, ketones, urobilinogen, bilirubin, blood (erythrocytes/hemoglobin), color, clarity) was performed, as well as a fecal examination (general fecal examination, test for parasites, lamblia, *Helicobacter pylori*, levels of zonulin and antitrypsin). Study participants were given a uniform procedure for collecting urine and stool samples on their own. After a plum-sized stool sample was gathered, it was placed in a tube and stored in a refrigerator (temp. 2–4° C) and then delivered to the laboratory within 24 h after the collection. The samples of urine and feces were submitted for testing to the laboratory at the same time as blood was collected.

Measurements were made using flow cytometry (blood count), kinetic-photometric method (ESR), indirect potentiometry (sodium, potassium, chloride), spectrophotometry (total protein, calcium, phosphorus, magnesium, total cholesterol, HDL, triglycerides, U, UA, iron, AST, ALT, creatinine, total bilirubin, GGTP, glucose), direct chemiluminescence (vitamin B_12_, vitamin D, ferritin), strip tests (general urinalysis), immunochromatographic method with Giardia test from Hydrex Diagnostics (*Giardia lamblia* in feces), and by H. pylori Ag by nal von minden (*Helicobacter pylori* in feces) and microscopically (general fecal examination and evaluation of food debris, testing for parasites, lamblia). All laboratory analyses described here were performed using the following equipment: Alinity HQ (Abbott, Singapore), Roller 20 (Alifax, Italy), Alinity C (Abott, Singapore), Alinity I (Abott, Singapore) and Atellica 1500 (Siemens, Germany) and were commissioned as an external service, performed by Alab Laboratories (Warsaw, Poland).

### Clinical event monitoring

Throughout each expedition, the climbers prospectively logged gastrointestinal (GI) complaints and symptoms suggestive of acute mountain sickness (AMS) in individual food diaries. Two participants reported transient diarrhea that was treated with a short course of oral nifuroxazide, and one additional episode occurred after returning to low altitude. One climber experienced AMS during the ascent. After an immediate descent to base camp, he received a single intramuscular dose of dexamethasone. No other antibiotics, proton pump inhibitors, or nonsteroidal anti-inflammatory drugs were taken during the study period.

### Aerobic and anaerobic performance testing

Aerobic capacity was assessed during a test with gradually increasing load performed until exhaustion, while anaerobic capacity was assessed during the Wingate test for the lower and upper limbs. Exercise tests were performed twice. The first test was performed about two weeks before the expedition, and the second test was performed up to four days (3.88 ± 1.49 days) after returning from the expedition at an altitude of about 383 m above sea level.

### Graded treadmill test

The test was performed on a mechanical treadmill h/p/Cosmos Saturn (h/p/Cosmos Sports & Medical GmbH, Germany) according to the following scheme: 2-minute recording of baseline cardiorespiratory indices in a standing position, a 4-minute run at a constant speed of 8 km/h with a treadmill angle of 1°, followed by a 1.1 km/h increase in running speed every 2 min until a running speed of 16.8 km/h was reached. In the remaining part of the test, the running speed did not change and an increase in exercise intensity was obtained by raising the angle of the treadmill by 1° every 2 min. The test continued until exhaustion, subjective to the test participant. Respiratory indices were monitored continuously using a Cortex Metalyzer 3B ergospirometer (CORTEX Biophysik GmbH, Germany), heart rate recorded using a Polar S-410 cardiac monitor (Polar-Electro, Finland).

### Wingate anaerobic test

This test was performed in a sitting position without getting up from the saddle on a Monark 894e cycloergometer for the lower limbs (Monark Sports & Medical, Sweden) and Monark 834e for the upper limbs (Monark Sports & Medical, Sweden) in a modified 20-second version. The participants’ task was to perform crank turns at a maximally high rhythm throughout the test. Both tests were preceded by warm-ups, with the lower limb test lasting 5 min with a load of 100 watts and the upper limb test lasting 4 min with a load of 60 watts. The pedaling rhythm was 60 rpm. During the warm-up for the lower limb test, brief 5–second accelerations of the pedaling rhythm to about 80% of subjective maximum capacity occurred in 2nd, 4th and 5th minute and in the warm-up for the upper limb test in 2nd and 4th minute, respectively. After the warm-up, the participants performed stretching exercises for 2 to 3 min, after which the test proper was started. The test load was set at 7.5% and 4.5% of body weight in the lower and upper limb tests, respectively.

### Assessment of lactate concentration

Before the start (5 min before exercise) and at the end of all tests (3 and 20 min after exercise), blood was drawn from the fingertip capillaries into tubes containing a glycolysis inhibitor to determine plasma lactate concentration. Lactate concentration was determined using LC2389 reagent (Randox Laboratories Ltd., UK) by an enzymatic colorimetric method, which allows the determination of biochemical markers by using a specific enzymatic reaction that yields a colored product. The concentration of lactate was determined by the color change of the solution, assessed by reading the absorbance at 550 nm using a Thermo Scientific Nicolet Evolution 201 PC Control dual-beam UV/VIS spectrophotometer (Thermo Fisher Scientific, USA). The absorbance results were recorded electronically, and were then used to calculate the concentration of lactate in each sample.

## Microbiome analysis

### Sample collection

Stool was collected and immediately placed in stool storage containers, and these were placed in a Styrofoam box with a cooling insert. The box with the stool sample was kept in the refrigerator (temp. 2–4° C) and then transferred within 24 h of collection to the project contractor. The stool was then placed in the freezer (temp. −80° C). The fecal samples were collected from participants in the same rounds as described in the section describing the health analysis (before and after the expedition).

### DNA isolation, quantitation and quantification

Approximately 200 mg of each frozen stool sample was processed for DNA isolation using the QIAamp PowerFecal Pro DNA Kit (Qiagen, Germany) following a modified protocol designed to enhance microbial cell disruption. This procedure combined physical, mechanical (bead-beating with a Bead Ruptor Elite; Omni International, USA), and chemical lysis steps. Homogenization involved short, high-speed cycles with cooling intervals. Key modifications included the following steps: after adding Solution CD1, the sample was incubated at 55 °C for 10 min with 500 rpm shaking and vortexing every 2 min. Further, bead beating was performed for 15 s at 6 m/s, repeated 4 times with a 2-minute break after each cycle, followed by a short spin. Next, 40 µl of proteinase K was added, and the mixture was incubated at 25 °C for 10 min. A final, 5th round of bead beating was performed for 15 s at 6 m/s. The extracted DNA was assessed for concentration and purity using a NanoDrop spectrophotometer (Thermo Scientific, USA), and the integrity of the DNA was verified on 1% agarose gels. Only the DNA samples with sufficient quality and yield were selected for further processing. The DNA concentration was then re-evaluated and standardized using a Qubit 3.0 Fluorometer (Thermo Fisher Scientific, USA) and the Qubit DNA BR assay, ensuring accurate normalization prior to library preparation.

### Library preparation and sequencing

Metagenomic libraries were prepared from 100 ng of input DNA per sample, using the KAPA HyperPlus Library Preparation Kit (Roche, Basel, Switzerland) as described in our previous papers^27,28^. The fragmentation step was optimized to produce fragments with an average size of 300–500 bp. Indexed adapter ligation was performed using KAPA Universal dual-indexed adapters at a controlled concentration to maintain efficient ligation and minimize adapter dimer formation. Libraries were then purified with KAPA Pure Beads (Roche, Switzerland), and library amplification was limited to six PCR cycles to reduce amplification bias. After PCR, a final clean-up step was performed to remove any residual primer dimers and non-target fragments. The resulting libraries were assessed for quality and fragment size distribution using a 4150 TapeStation system (Agilent Technologies, USA).

All libraries were then normalized to 20 nM, pooled in equimolar amounts, and subjected to 150 bp paired-end sequencing on an Illumina NovaSeq platform (Illumina, CA, USA). Each library generated approximately 13 million paired-end reads. This deep sequencing strategy provided a comprehensive snapshot of the microbial communities and their genomic characteristics.

### Statistics and bioinformatics analysis

The raw fastq sequences were subjected to quality control with TrimGalore version 0.6.10^29^. Three samples were rejected from further analysis due to poor reading quality. To remove human contaminants, any reads mapping to the human reference genome build 38 with bowtie2 version 2.2.3^30^ were removed. Taxonomic and functional profiles were calculated via MetaPhlAn 4.0 and HumanN 3.7^31^. Differential enrichment analysis was performed using the Linear Discriminant Analysis Effect Size analysis (LEfSe)^32^. The LEfSe analysis was implemented in Python, leveraging functions from the scipy.stats module (version 1.11.1) for non-parametric statistical tests, specifically kruskal for identifying features with significant differential abundance. Subsequently, sklearn.discriminant_analysis.LinearDiscriminantAnalysis (Scikit-learn version 1.3.0) was utilized to calculate the Linear Discriminant Analysis (LDA) score, which estimates the effect size of each differentially abundant feature. Alpha diversity (Shannon, Simpson, species richness) was calculated using the scipy module (version 1.11.1). Beta diversities (Bray-Curtis) were calculated using the QIIME 2^33^ amplicon-2024.2 version diversity packages and the PERMANOVA test was used to identify statistical significance. Finally, to investigate relationships between microbiome features and metadata variables, Spearman’s rank correlation coefficients were calculated. This analysis was performed using the spearmanr function from the scipy.stats module (version 1.11.1). To control for the false discovery rate arising from multiple comparisons, the resulting p-values were adjusted using the Benjamini-Hochberg (BH) correction. This multiple testing correction was applied via the multipletests function from the statsmodels.stats.multitest submodule, part of the statsmodels package (version 0.14.1)^34^. The entire analysis was conducted within a Python 3.11.9 environment and visualized using the matplotlib 3.7.2 and seaborn 0.12.2 packages.

## Results

### Cohort overview

In this study, we investigated 17 experienced Polish male mountaineers (23–40 years old) who undertook high-altitude expeditions lasting more than three weeks at altitudes above 3,000 MASL. Before and after their expeditions, we collected comprehensive data including dietary intake, blood and urine parameters, aerobic and anaerobic performance measures, and detailed metagenomic analyses of their gut microbiomes. By integrating these multidimensional datasets, our goal was to characterize how exposure to extreme mountain environments and dietary changes influence gut microbial community structure, functional pathways, and potential health and performance outcomes.

### Clinical events during the expeditions

Two participants experienced self-limited diarrhea that resolved within 48 h after taking nifuroxazide orally. One climber reported diarrhea upon returning to a lower altitude. One participant developed acute mountain sickness, which was managed by descending to base camp and administering a single intramuscular dose of dexamethasone. No additional gastrointestinal complaints were recorded.

### Microbiome changes after the mountaineering expedition

**Fig. 1:**
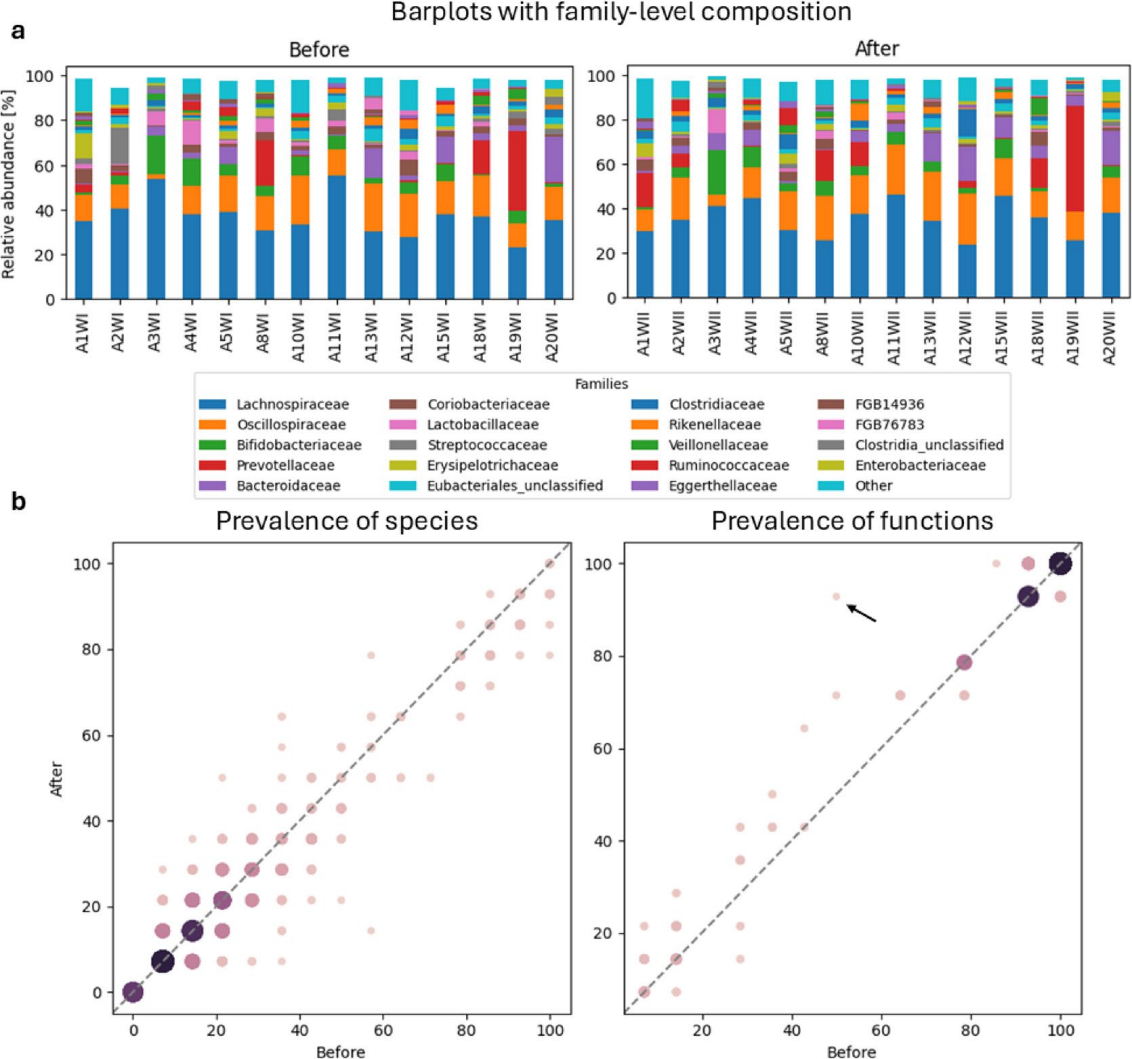
Comparison of gut microbiome compositions before and after the expedition. (**a**) Barplots with family-level composition of each sample before and after the expedition. (**b**) Prevalence of species (left) and functions (right) before versus after the expedition. The *GLUCOSE1PMETAB-PWY*: *glucose and glucose-1-phophate degradation pathway*, which substantially changed in prevalence as a result of the travel, is marked with a black arrow.

All participants exhibited generally diverse microbiomes both before and after the expedition (Fig. 1a, Supplementary Fig. 1). While the relative abundance of the most plentiful species did not change much in some individuals (A3), others experienced greater shifts in the microbiome (A10). There were on average 200 ± 69 bacterial species identified per participant before and 179 ± 53 species per participant after the expedition. In contrast, we found 76 ± 8 bacterial functions per participant before and 77 ± 8 functions after the expedition. The differences in the numbers of species and functions before and after the expedition were not significant.

Across all participants, the prevalence of species remained stable after the travel (Fig. 1b, left). This was particularly true for species occurring with low prevalence (present in less than a third of individuals), and any differences could be attributed to changes in only up to two individuals. We observed a slightly different trend in the comparison of functions - this time, the most stable functions were highly prevalent. There was one outlier, however; it was the *GLUCOSE1PMETAB-PWY: glucose and glucose-1-phosphate degradation* pathway. This function, present in a half of individuals before the expedition, was found in almost everyone upon their return. We observed a statistically significant increase in the abundance of this function as a result of the expedition (Wilcoxon signed-rank test, *p* < 0.05).

**Fig. 2:**
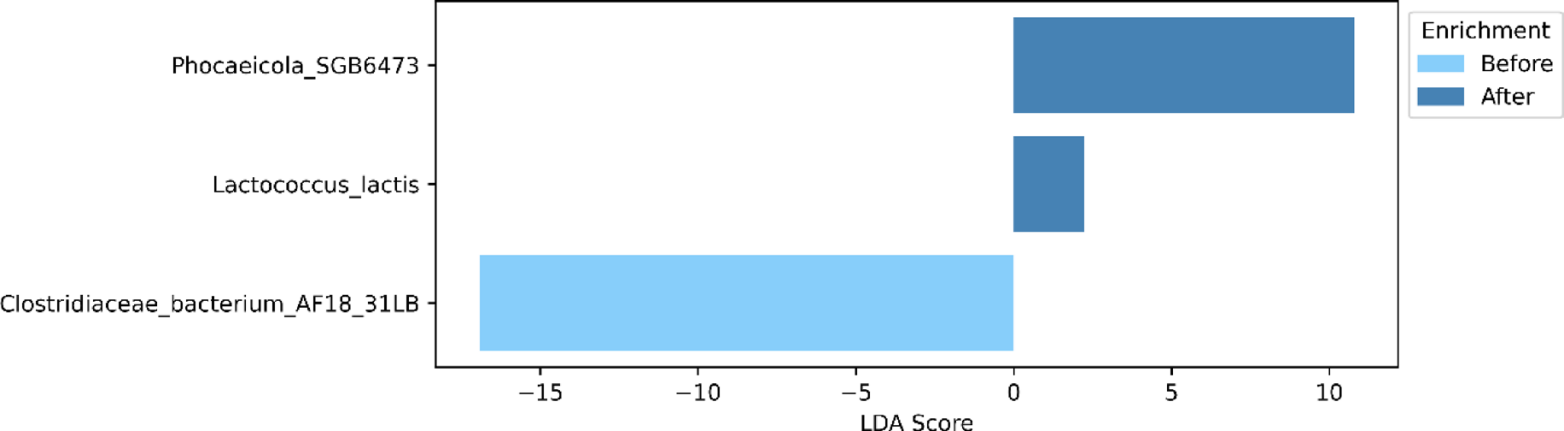
Species enriched in the participants before and after the expedition.

A differential enrichment analysis performed using LEfSe revealed two species enriched after the expedition and one before (Fig. 2). The two species which increased in abundance after the expedition were *Lactococcus lactis* and a species belonging to the *Phocaeicola* genus. On the other hand, there was a depletion trend of the *Clostridiaceae* bacterium. We found no statistically significant alterations in microbial functions.

Correlating microbiome features with dietary information revealed an association of a *PWY-6703: preQ0 biosynthesis pathway* with vitamin B_6_ and C intake before the expedition (Spearman correlations of 0.83 and 0.86, respectively, adjusted p-values < 0.05). We observed a decrease in the intake of the vitamins after the expedition, which was significant for vitamin C (vitamin B_6_: 6.1 mg to 2.6 mg, T-test p-value > 0.05; vitamin C: 325.7 mg to 149.2 mg, T-test p–value < 0.01). Similarly, the average abundance of the *PWY-6703: preQ0 biosynthesis pathway* showed a notable, but statistically insignificant decrease (from 18% to 15%). No other associations of functions or species with dietary parameters before or after the expedition passed multiple testing corrections.

### Varying degrees of Microbiome alterations among participants

**Fig. 3:**
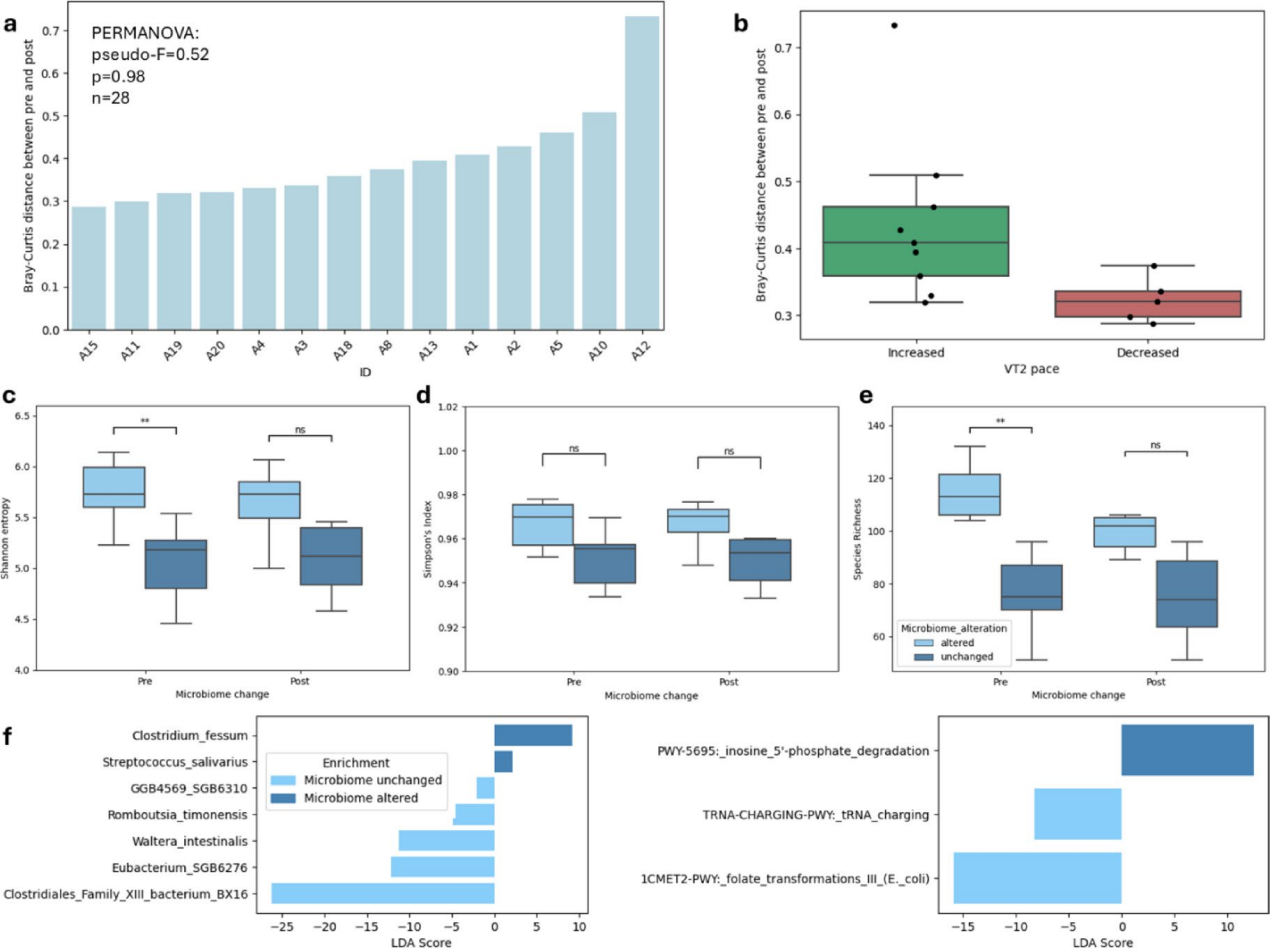
A comparison of participants with greater and smaller gut microbiome alterations after the high-altitude travel. (**a**) Bray-Curtis distance between matched samples (before and after the expedition) from the same participant. (**b**) Individuals who observed an improvement in the VT2 pace after the expedition experienced greater microbiome alterations. (**c**),(**d**),(**e**) The microbiome of individuals who experienced greater microbiome alterations was significantly richer before the expedition. (**f**) LEfSe differential enrichment analysis comparing species and functions in individuals with and without large microbiome alterations at baseline.

Following our observation that the microbiomes were altered in individuals to different degrees (Fig. 1a), we calculated Bray-Curtis distances between matched samples from the same individual, collected before and after the intervention (Fig. 3a, Supplementary Fig. 2). We observed a range of distances between the samples, with samples from the individual A12 being visibly more differentiated than the rest.

Separating the individuals based on their second ventilatory threshold (VT2) pace change after the expedition revealed that individuals who experienced an improvement in fitness experienced a notably yet not significantly greater microbiome alteration (Fig. 3b). In addition, participants whose microbiomes changed more (Bray-Curtis distance ≥ 0.36, chosen based on the median) had significantly richer microbiomes before the intervention in comparison to those who experienced subtle shifts (Fig. 3c-e). This was accompanied by insignificant but notably higher intakes of manganese, calcium, vitamin A, magnesium and phosphorus (mean manganese intake 9.2 and 3.9 mg, calcium 1520.7 and 973.3 mg, vitamin A 2246.0 and 1291 µg, magnesium 687.7 and 397.6 mg, phosphorus 2021.5 and 1545.7 mg in participants with and without substantial gut alterations, *p* < 0.05, adjusted p-values > 0.05).

A LEfSe differential enrichment analysis of species and functions at baseline revealed statistically significant differences between the two groups (Fig. 3f). The microbiomes of individuals who experienced alterations were enriched in *Clositridium fessum*, *Streptococcus salivarius* and the *inosine 5’-phosphate* *degradation pathway*. On the other hand, they appeared to have lower abundances of, among others, a bacterium from the Clostridiales family as well as the *folate transformations* and *tRNA charging* *pathways*.

### Microbiome correlations with blood markers, anaerobic capacity physiological markers and diet in participants exhibiting the strongest Microbiome shifts

**Fig. 4:**
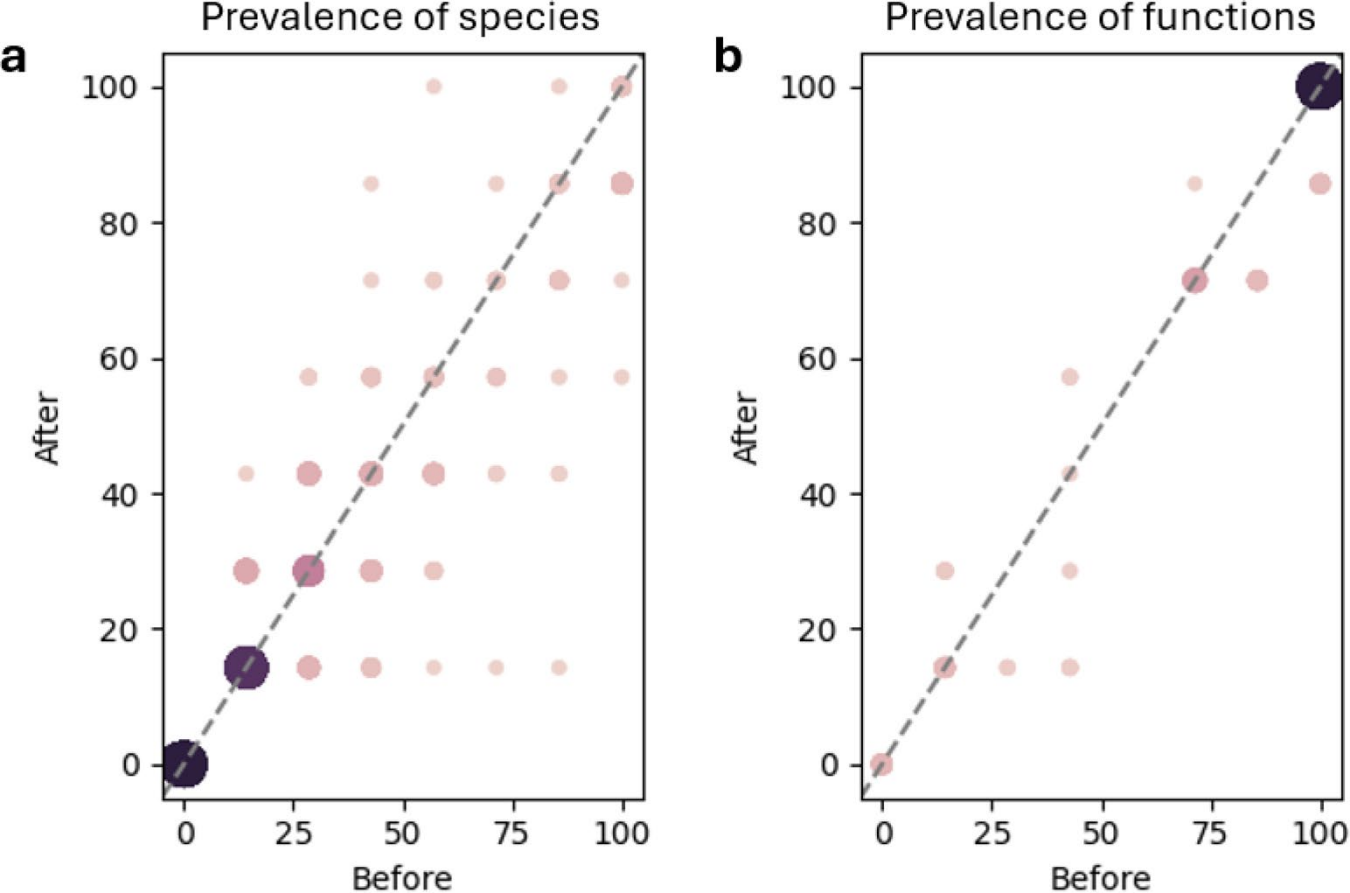
Prevalence comparison of species (**a**) and functions (**b**) in individuals with large microbiome alterations as a result of the expedition.

Investigating the subset of individuals with large microbiome alterations revealed shifts in prevalence of species were more varied than that of functions (Fig. 4a and b). The taxa with the greatest alterations included *Dorea sp AF36_15AT*, present in 57% of individuals before and in 100% after the expedition, as well as *GGB9758_SGB15368* from the Firmicutes phylum, found in 43% before and 86% of individuals after. While the prevalence of functions remained relatively stable, with most functions being present both before and after the expedition, a few functions experienced a substantial prevalence drop. They were *tetrapyrrole biosynthesis I (from glutamate)*,* UDP-N-acetyl-D-glucosamine biosynthesis I* and *O-antigen building blocks biosynthesis (E.coli)*, all present in 43% before and 14% of individuals after the expedition.

A differential enrichment analysis with LEfSe identified 3 species, namely *Blautia luti*, *Romboutsia timonensis* and *GGB4569_SGB6310* from the Lactobacillaceae family, enriched in abundance after the expedition. We found no species enriched before, as well as no statistically significant alterations of abundance in functions.

We identified several microbiome feature correlations with blood markers and anaerobic indices (Table 2). Before the expedition, there were two species associated with potassium, specifically *Prevotella copri clade A* (positive correlation) and *Bacteroides uniformis* (negative correlation), as well as 15 cases of marker associations with microbial functions. *dTDP-β-L-rhamnose biosynthesis pathway* was the only function associated with two different markers before the expedition, specifically total work performed of lower limbs (W_t_) and mean anaerobic power of lower limbs (P_mean_) (positively in both cases). After the investigation, we found one association with species *Agathobaculum butyriciproducens* with calcium (strong positive correlation) and 8 with functions. This time, two functions were associated with at least two markers (*NAD de novo biosynthesis I (from aspartate)* positively with basophils and calcium; and *superpathway of L-serine and glycine biosynthesis I* negatively with sodium and positively with cholesterol HDL). Finally, two functions had different associations both before and after the expedition. G*uanosine ribonucleotides de novo biosynthesis* was positively correlated with sodium before and negatively with mean platelet volume (MPV) after, while *myo-*,* chiro- and scyllo-inositol* *degradation* was positively associated with sodium before and with basophils after. Other analyzed correlations of microbiome features with blood, urine, fecal markers and anaerobic indices were not statistically significant.

**Table 2:** Statistically significant correlations (adjusted p-values < 0.05) of blood markers and anaerobic indices with Microbiome features in individuals with Microbiome alterations, before and after the expedition (*n* = 7).

Timepoint	Parameter	Microbiome feature	Spearman correlation
Before	Potassium [mmol/l]	*Prevotella copri clade A*	0.98
		*Bacteroides uniformis*	−0.97
	LA (20’) [mmol/l]	*GGB9642 SGB15119*	−0.99
	HGB [g/dl]	*RIBOSYN2-PWY: flavin biosynthesis I (bacteria and plants)*	0.96
	Sodium [mmol/l]	*ANAGLYCOLYSIS-PWY: _glycolysis_III_(from_glucose)*	0.94
		*GLUTORN-PWY: L-ornithine biosynthesis I*	0.94
		*PWY-1042: glycolysis IV*	0.94
		*PWY-2942: L-lysine biosynthesis III*	0.94
		*PWY-5097: L-lysine biosynthesis VI*	0.94
		*PWY-7221: guanosine ribonucleotides de novo biosynthesis*	0.94
		*PWY-7237: myo-*,* chiro- and scyllo-inositol degradation*	0.94
	HDL [mmol/l]	*ARGSYN-PWY: L-arginine biosynthesis I (via L-ornithine)*	−0.96
		*ARGSYNBSUB-PWY: L-arginine biosynthesis II (acetyl cycle)*	−0.96
	W_t_ [J ∙ kg^−1^]	*DTDPRHAMSYN-PWY: dTDP-&beta;-L-rhamnose biosynthesis*	0.96
	P_mean_ [W ∙ kg^−1^]	*DTDPRHAMSYN-PWY: dTDP-&beta;-L-rhamnose biosynthesis*	0.96
	t_m_ [s]	*PWY0-1296: purine ribonucleosides degradation*	0.96
	WSHL [W/kg/s]	*ILEUSYN-PWY: L-isoleucine biosynthesis I (from threonine)*	0.96
After	Calcium [mmol/l]	*Agathobaculum butyriciproducens*	−0.99
	MPV [fL]	*PWY-7221: guanosine ribonucleotides de novo biosynthesis*	−0.96
	Basophils [10^9^/L]	*PWY-7237: myo-*,* chiro- and scyllo-inositol degradation*	0.96
		*PYRIDNUCSYN-PWY: NAD de novo biosynthesis I (from aspartate)*	0.96
	Sodium [mmol/l]	*SER-GLYSYN-PWY: superpathway of L-serine and glycine biosynthesis I*	−0.96
	Calcium [mmol/l]	*PYRIDNUCSYN-PWY: NAD de novo biosynthesis I (from aspartate)*	0.99
	Magnesium [mmol/l]	*PWY-6151: S-adenosyl-L-methionine salvage I*	−0.98
	HDL [mmol/l]	*SER-GLYSYN-PWY: superpathway of L-serine and glycine biosynthesis I*	0.96
	Protein total [g/l]	*HISTSYN-PWY: L-histidine biosynthesis*	0.96

Abbreviations: HDL – high-density lipoprotein cholesterol; HGB – haemoglobin; LA (20’) – lactate concentration at intake at 20 min after aerobic capacity test; MPV - mean platelet volume; P_mean_ – mean anaerobic power (lower limbs); t_m_ – time of maintaining maximum power (lower limbs); WSHL - rate of decrease from the maximum power of the lower limbs; W_t_ – total work performed (lower limbs).

Finally, we found a number of microbial function associations with diet after the expedition (all of which passed the correction for multiple testing, Supplementary Table 1). We identified strong positive correlations (Spearman correlation > 0.90) of *coenzyme A biosynthesis* with overall calorie and carbohydrate intake, monounsaturated fatty acid (MUFA) as well as vitamins C, B_12_, B_1_ and B_6_. *L-arginine biosynthesis* was associated with overall carbohydrate intake and digestible carbohydrates, while the *penthose phosphate pathway* with selenium and plant protein with *sucrose biosynthesis*. Negative correlations were found between *adenine and adenosine salvage* and sodium, as well as between *L-rhamnose biosynthesis* and animal protein.

## Discussion

### Microbiome and nutritional adaptations to High-Altitude stress

The observed changes in gut microbiome compositions before and after the high-altitude mountaineering expedition indicate adaptive shifts in the gut microbiome and host physiology in response to extreme environmental and physiological stressors. Our findings demonstrate that exposure to low atmospheric pressure, hypoxic conditions, and challenging dietary changes can reshape the gut microbiome, potentially affecting host metabolism, immune function, and overall performance. Similar to previous studies showing that altitude-induced hypoxia alters energy metabolism and physiological adaptations in athletes^35–37^, we identified functional shifts in the gut microbiome, such as increased prevalence of the *glucose and glucose-1-phosphate degradation* *pathway*, which was present in half of individuals before the expedition and in almost all participants upon their return. In addition, the abundance of this pathway increased significantly. The presence of this pathway likely reflects not only the elevated intake of simple carbohydrates during the expedition - through consumption of energy gels, sweetened bars, isotonic drinks, and freeze-dried meals^38,39^ - but also an adaptive microbial response to environmental and physiological stressors, including hypoxia and increased energy demand. While the specific *glucose and glucose‑1‑phosphate degradation pathway* has not been directly reported in earlier hypoxia studies, a number of investigations in altitude-adapted populations suggest broader functional shifts in microbial carbohydrate metabolism under environmental stress^40–42^. The observed increase in the prevalence of the *glucose and glucose-1-phosphate degradation pathway* may indicate both an increase in substrate availability and a microbiome strategy to maximize ATP yields in the presence of restricted oxygen.

From a nutritional perspective, our results highlight the importance of vitamin intake, particularly vitamins C and B_6_, in modulating microbial metabolic pathways such as the *preQ0 biosynthesis* *pathway*. The observed association between the *preQ0 biosynthesis pathway* and dietary intake of vitamins B_6_ and C prior to the high-altitude mountaineering expedition highlights the intricate interplay between nutrition, microbiome dynamics, and metabolic adaptation. Vitamin B_6_, known for its role as a cofactor in amino acid metabolism^43^, is essential for the synthesis of nucleotides and neurotransmitters. This vitamin may enhance the efficiency of metabolic processes involved in preQ0 production, as the pathway is likely reliant on amino acid precursors. Additionally, vitamin C serves as a potent antioxidant, crucial for mitigating oxidative stress that can be exacerbated in high-altitude environments^44–46^. Its antioxidant properties may also support beneficial gut microbial populations capable of metabolizing this vitamin and subsequently influencing pathways like the *preQ0 biosynthesis pathway*. The dietary intake of these vitamins may modulate gut microbiome composition, ensuring the availability of substrates necessary for microbial metabolism, which in turn supports the *preQ0 biosynthesis pathway*. This association underscores the significance of adequate vitamin intake in preparing the body for the metabolic demands of high-altitude exposure, where increased energy expenditure and altered oxygen availability necessitate robust cellular adaptations^47^.

An analysis of Bray-Curtis distances between baseline and post-expedition samples revealed varying degrees of microbiome alterations among the participants. Individuals with greater microbiome shifts responded to the high-altitude conditions better through improvements in fitness markers such as a higher pace at the VT2. This observation suggests that greater microbiome plasticity might be associated with improved metabolic or cardiorespiratory adaptation under environmental stress, such as hypobaric hypoxia^19,48^. Interestingly, participants with larger microbiome shifts also had significantly richer and more diverse baseline microbiomes. Greater microbial diversity has been previously linked with enhanced resilience and adaptive capacity of the gut ecosystem, potentially allowing for more dynamic functional reorganization in response to stressors like high altitude, changes in diet, or physical exertion^49–51^. This was accompanied by insignificant but notably higher intakes of macronutrients, such as manganese, calcium, vitamin A, magnesium and phosphorus before the expedition, which have been implicated in supporting microbial diversity, intestinal barrier integrity, and immune function^52–56^. Finally, their microbiomes were enriched in *Clostridium fessum*, *Streptococcus salivarius* and the *inosine 5’-phosphate degradation pathway*. *S. salivarius* has been identified as a commensal species with immunomodulatory properties and the ability to produce bacteriocins and anti-inflammatory compounds^57,58^, while *Clostridium fessum* may contribute to short-chain fatty acid production, although its role remains less well understood. SCFAs (e.g., acetate, propionate, butyrate) are key microbial metabolites derived from fermentation of dietary fibers and play important roles in host energy metabolism, immune regulation, and gut barrier integrity^59^. The presence of the *inosine 5’-phosphate degradation pathway* may reflect increased nucleotide turnover and energy metabolism potential, which has been observed in microbiomes adapting to metabolic stress^60^. These preliminary findings suggest that baseline microbiome composition and its capacity to dynamically respond to environmental stressors may influence the physiological outcomes of high-altitude exposure. Future studies with larger cohorts are needed to explore these associations further and confirm causality. A similar performance-oriented plasticity is emphasized in our recent narrative review of athletic gut microbiomes^61^, which shows that elite performers consistently harbour SCFA- and bile-acid-producing consortia that safeguard energy supply, immunity and barrier function during heat, hypoxia and prolonged exertion - paralleling the altitude-driven shifts observed here.

Importantly, several microbial features that differed within our cohort showed significant correlations with physiological read-outs, suggesting that the compositional shifts we observed may have functional - rather than purely cosmetic - consequences. Before the expedition, several microbiome-host links converged on the same theme: a *Prevotella*-rich, rhamnose-active community was associated with superior lower-limb power and tighter electrolyte control. Specifically, the *dTDP-β-L-rhamnose-biosynthesis pathway* showed a strong positive correlation with both total work and mean anaerobic power of the lower limbs (ρ = 0.96 for each; Table 2), while serum potassium related positively to *Prevotella copri* (ρ = 0.98) and negatively to *Bacteroides uniformis* (ρ = −0.97). Taken together, these baseline patterns indicate that athletes entering the expedition with a *Prevotella*-rich enterotype may enjoy more efficient mineral absorption and rapid carbohydrate utilisation^19^ - an interpretation consistent with endurance cohorts in which *Prevotella* enrichment tracks high-carbohydrate, fibre-dense diets and long weekly training volumes^62^. In our climbers, the same triad - *P. copri*, elevated potassium, and the rhamnose nucleotide-sugar route - suggests a microbiome “fitness reserve” that supports energy flux and electrolyte stability when oxygen becomes limited at altitude.

Consistent with the idea that pre-existing gut configurations modulate later physiological responses, our recent case-control study in collegiate men performing maximal Wingate and Bruce tests revealed a different, but conceptually similar, pattern: baseline enrichment of *Clostridium phoceensis* and *Catenibacterium* spp. predicted blunted IL-1α and TIMP-1 responses to the aerobic (Bruce) protocol, whereas anaerobic testing elicited distinct microbiome–cytokine couplings driven by ethanolamine- and pyrimidine-related pathways^28^. After the climb, a distinct post-expedition signature emerged. Enrichment of the *NAD*^+^*de-novo-biosynthesis I pathway* correlated positively with circulating calcium (ρ = 0.99) and with basophil counts (ρ = 0.96), whereas the *super-pathway of L-serine/glycine biosynthesis* related inversely to sodium (ρ = − 0.96) but positively to HDL cholesterol (ρ = 0.96). In parallel, the *myo-*,*chiro- and scyllo-inositol degradation pathway* also tracked with basophils (ρ = 0.96), and *guanosine-ribonucleotide biosynthesis* showed an inverse relationship with mean platelet volume (ρ = − 0.96). Post-expedition functional read-outs therefore realigned earlier associations: at baseline both *guanosine-ribonucleotide biosynthesis* and the *myo-/chiro-/scyllo-inositol degradation pathway* tracked positively with serum sodium, whereas after altitude exposure the same routes coupled to MPV and basophils, respectively. This directional reversal underscores a flexible microbiome contribution to nutrient flux, electrolyte balance and immune signalling under chronic hypoxia. Taken together, these links connect post-expedition shifts in nucleotide, redox-cofactor and amino-acid metabolism to haematological (basophils, MPV), electrolyte (sodium, calcium) and lipid (HDL) read-outs, supporting a role for the gut microbiome in coordinating metabolic and immune adaptation under prolonged hypoxia and high energy demand^63^. Importantly, intestinal-permeability markers (zonulin and α₁-antitrypsin) remained unchanged, consistent with recent reports that well-acclimatized alpinists can maintain epithelial integrity under hypobaric stress^8^.

A differential enrichment analysis with LEfSe in our study identified 3 species, namely *Blautia luti*, *Romboutsia timonensis* and *GGB4569_SGB6310*, enriched in abundance after the expedition. Similarly, Su et al. showed a relative and absolute increase in *Blautia A* abundance in men who were exposed to high altitudes (> 3,600 MASL) for more than 2 months^41^. *Blautia A* species were shown to be involved in a module of “cobalamin biosynthesis” and butyric acid production that are both beneficial to microbial ecosystems and intestinal epithelial cells^41,64^. An in vivo animal experiment supported a crucial role of *Blautia A* species in facilitating host fitness to hypoxia environments, likely via anti-inflammation and intestinal barrier protection to maintain intestinal health, thereby suggesting a high translational potential of *Blautia A* species as a candidate probiotic agent for the prevention or treatment of hypoxia-associated maladaptation or disorders. Taken together, these observations of increased abundance of *Blautia A* imply this genus is a beneficial high-altitude bacterial group that may play an important role in promoting acclimatization and adaptation to high-altitude conditions^41^.

We observed significant microbial functional associations with dietary intake after the expedition. Notably, strong positive correlations with *coenzyme A biosynthesis* and various dietary components suggest that these nutrients may enhance microbial metabolic pathways essential for energy production and lipid metabolism. Similarly, positive associations of *L-arginine biosynthesis* with overall carbohydrate intake and the assimilation of carbohydrates indicate that dietary carbohydrates may support microbial synthesis of this amino acid, which is crucial for various physiological functions^18,19,63^. Furthermore, associations of the *pentose phosphate pathway* with selenium and plant protein with sucrose biosynthesis highlight the potential role of specific micronutrients and macronutrients in modulating microbial metabolic pathways. On the other hand, negative associations of *adenine and adenosine salvage* pathways with sodium intake, as well as between *L-rhamnose biosynthesis* and animal protein consumption could suggest competitive inhibition or substrate limitation in these metabolic processes. Overall, the results emphasize complex interactions between dietary components and microbial metabolic activities in the gut. However, the sample size for this analysis was limited (*n* = 7); thus, future validation of these findings in a larger cohort is necessary.

### Practical Implications, Limitations, and future perspectives

From an applied perspective, these findings provide valuable guidance for mountaineers, coaches, and sports nutritionists. Pre-expedition strategies might include optimizing micronutrient status, particularly for vitamins and minerals shown here to correlate with beneficial microbial functions and performance markers. During expeditions, integrating nutrient-dense, microbiome-supportive foods – even if partially in the form of supplements or freeze-dried products – may help maintain beneficial gut microbiota profiles and associated metabolic functions. Beyond the athletic context, our findings contribute to a growing body of literature on the role of gut microbiome in adaptation to extreme environments^40,41,65–69^, highlighting opportunities to leverage nutritional interventions to support both health and performance.

Climbers should avoid taking non-prescribed antibiotics when possible, as agents such as nifuroxazide can temporarily reduce gut microbial diversity. Gradual acclimatization, along with a diet rich in fiber, polyphenols, and adequate carbohydrates, may further support gut barrier integrity and mitigate AMS-related inflammation^61^.

Future research should aim to confirm these findings in larger cohorts and explore targeted interventions-such as prebiotics, probiotics, postbiotics, or tailored micronutrient supplementation to promote a beneficial gut microbiome response in high-altitude environments. Integrating metagenomics with host transcriptomics, metabolomics, and immune profiling, as well as conducting controlled feeding studies, could further elucidate the mechanisms linking the gut microbiome, diet, and physiological adaptation in extreme sports and beyond.

Our study has some limitations. The climbers did not constitute a single group, led by a single climbing goal in the territory of a single country and mountain range. Climbers, characterized by the characteristics described in the study, constitute a small but elite group in Poland, hence several distinct expedition groups, climbing at similar altitudes and with similar sporting goals, but in different parts of the world, qualified for the study. Gathering and encouraging participants was one of the most challenging stages of the project. While all expeditions involved high-altitude exposure above 3000 MASL, other factors such as temperature, humidity, and radiation levels were not controlled for and may have contributed to interindividual differences in microbiome responses. Future studies conducted in more standardized settings are warranted to disentangle the specific environmental drivers of microbiome changes at altitude.

Due to the small number of GI disturbance (*n* = 3) and AMS (*n* = 1) events, the study lacked the power to detect reliable microbiome correlates. Exploratory diversity and taxonomic scans did not reveal outliers (see Supplementary Fig. 3). Likewise, exploratory alpha- and beta-diversity metrics and LEfSe screening (species and MetaCyc pathways, *p* < 0.05) failed to identify discriminant features. Larger cohorts are needed to clarify any causal links.

In addition to the level of hypoxia, other factors such as nutrition and the type of physical activity performed in the mountains, as well as the number of active days versus the number of rest days, may contribute significantly to the observed changes in microbiome. Climbing groups active in Peru could have used the downtime between several days of mountain activity to descend to a town at the foot of the mountains to compensate for the negative energy balance there, while expedition groups in Kyrgyzstan, Nepal or Pakistan were dependent on the food they took into the mountains or that the expedition agency offered them.

We acknowledge that some participants consumed sports supplements and functional foods for athletes, which - according to Álvarez-Herms et al.^23^ - may influence gut microbiota composition and gut barrier integrity in athletes. Although supplement intake was recorded in our study, we did not specifically analyze the potential impact of additives commonly found in these products (e.g., emulsifiers, artificial sweeteners, preservatives) on the gut microbiota. Furthermore, we did not observe a clear association between general supplement use and microbiome shifts or performance outcomes. Nevertheless, considering the small sample size, individual variability, and heterogeneous supplement composition, we cannot fully exclude a modulatory effect. Future studies should incorporate detailed assessments of supplement ingredients, including non-nutritive additives, to better understand their influence under high-altitude stress conditions.

## Conclusions

In conclusion, our study demonstrates that the gut microbiome is responsive and potentially malleable to the dietary and environmental stresses encountered during high-altitude mountaineering. The observed changes in microbial composition and function, their associations with micronutrient intake, and their potential links to improved athletic performance underscore the integral role of the microbiome as an adaptive partner in extreme human endeavors^61^.

## Supplementary Information

Below is the link to the electronic supplementary material.

Supplementary Material 1

## Data Availability

The raw fastq sequences have been deposited to the European Nucleotide Archive (ENA) under the accession PRJEB84244.
